# Assessing axonal pathology and disease progression in chronic inflammatory demyelinating polyneuropathy using corneal confocal microscopy

**DOI:** 10.1007/s00415-024-12812-4

**Published:** 2024-12-12

**Authors:** Rafael Klimas, Dietrich Sturm, Annika Altenborg, Nayia Stylianou, Sophie Huckemann, Zornitsa Gasz, Thomas Grüter, Jörg Philipps, Tineke Greiner, Christoph Maier, Lynn Eitner, Elena Enax-Krumova, Matthias Vorgerd, Peter Schwenkreis, Ralf Gold, Anna Lena Fisse, Jeremias Motte, Kalliopi Pitarokoili

**Affiliations:** 1 Department of Neurology, St. Josef-Hospital, Ruhr-University, Gudrunstrasse 56, 44791 Bochum, Germany; 2 Immunmediated Neuropathies Biobank (INHIBIT), Ruhr-University, Bochum, Germany; 3 Department of Neurology, Agaplesion Bethesda Hospital, Wuppertal, Germany; 4 Department of Neurology, BG University-Hospital Bergmannsheil Bochum, Ruhr-University, Bochum, Germany; 5 Department of Neurology and Stroke Unit, Evangelical Hospital Lippstadt, Lippstadt, Germany; 6 Department of Neurology and Neurogeriatrics, Johannes-Wesling-Klinikum Minden, Ruhr-University, Bochum, Germany; 7 Department of Pediatrics, St. Josef-Hospital, Ruhr-University Bochum, Bochum, Germany

**Keywords:** CCM, CIDP, Immunneuropathies, Polyneuropathies

## Abstract

**Objective:**

Chronic inflammatory demyelinating polyradiculoneuropathy (CIDP) is an autoimmune neuropathy characterized by progressive or relapsing–remitting weakness and sensory deficits. This study aims to evaluate the utility of corneal confocal microscopy (CCM) in diagnosing and monitoring CIDP.

**Methods:**

We analysed 100 CIDP patients and 31 healthy controls using CCM to measure corneal nerve fiber density (CNFD), length (CNFL), and branch density (CNBD). Standardized clinical and electroneurographic evaluation were conducted, and statistical analyses were performed to compare CCM parameters between groups and across disease stages.

**Results:**

CIDP patients and subgroups exhibited significant reduction in CNFD, CNFL, and CNBD compared to controls. This reduction was observed in late disease stages and severe overall disability sum score (ODSS), and Inflammatory Neuropathy Cause and Treatment Sensory Sum Score (ISS). CCM parameters correlated with axonal pathology in electroneurography of sensory, but not motor nerves. Despite the significant differences, the diagnostic sensitivity (41%) and specificity (77%) of CCM parameters were limited.

**Conclusion:**

While CCM effectively differentiates CIDP patients from healthy controls and was associated with disease severity, its diagnostic accuracy for routine clinical use is a posteriori. However, CCM shows promise as a non-invasive tool for monitoring sensory axonal pathology in CIDP.

**Supplementary Information:**

The online version contains supplementary material available at 10.1007/s00415-024-12812-4.

## Introduction

Despite established criteria and technical innovations, the diagnosis and monitoring of chronic inflammatory demyelinating polyradiculoneuropathy (CIDP) still remains challenging. CIDP represents the most prevalent chronic form of autoimmune neuropathy. Characterized by progressive or relapsing–remitting weakness and sensory deficits, CIDP exhibits a population prevalence ranging from 0.8 to 8.9 per 100,000 individuals [1, 2]. Despite recent advancements, the underlying pathogenic mechanisms of CIDP remain incompletely elucidated. It likely involves a complex interplay between cellular and humoral immune responses, leading to demyelination and axonal injury [3, 4].

Diagnosing CIDP presents a significant challenge due to the heterogeneity of its clinical presentation and the absence of definitive diagnostic and biological markers [3]. Current diagnostic criteria rely on a combination of clinical features and electrophysiological studies alongside with the exclusion of alternative causes of neuropathy [5–7]. However, these established criteria may not always provide sufficient evidence for a conclusive diagnosis, particularly in cases of advanced neurodegenerative changes and the differentiation of typical and variant disease entities [3, 8].

Corneal confocal microscopy (CCM) is a non-invasive technique that enables in vivo microscopic examination of the cornea. Previous studies have shown that corneal nerve fiber density (CNFD), corneal nerve fiber length (CNFL), and corneal nerve branch density (CNBD) are reduced in immune-mediated neuropathies [9], as well in many other neuropathies with different etiologies [10]. In contrast, corneal inflammatory cells (CIC) are increased in patients with CIDP, which has been associated with disease progression [11, 12]. Therefore, CCM has the potential to be an early indicator of impending disability in chronic immune neuropathies, helping physicians to make treatment decisions. However, the current research on the significance of CCM in relation to neuropathies is not yet sufficiently valid for clinical routine.

This study aims to investigate the diagnostic and disease-monitoring potential of CCM in patients with CIDP, comparing corneal nerve parameters and their correlation with clinical and electrophysiological findings. By evaluating the sensitivity and specificity of CCM, we aim to determine its utility in clinical practice for managing CIDP.

## Methods

### Patients and healthy controls

100 patients with CIDP were analysed between January 2018 and October 2023. Patients were recruited from the INHIBIT registry, established in 2019 in our institution and underwent standardized clinical and paraclinical evaluation. They were diagnosed in accordance with the diagnostic criteria of the European Federation of Neurological Societies/Peripheral Nerve Society (EFNS/PNS) [5]. 31 subjects without diagnosis of polyneuropathy, diabetes or alcohol abuse served as healthy controls (HC). Data collection included sociodemographic data (age, sex, date of first manifestation, date of diagnosis), specific diagnosis (typical CIDP, atypical CIDP, distal acquired demyelinating symmetric polyneuropathy (DADS), multifocal acquired demyelinating sensory and motor neuropathy (MADSAM), focal CIDP, pure motor CIDP, and pure sensory CIDP), clinical scores (see below), nerve conduction studies (NCS, see below) and CCM measurements (see below).

### Clinical scores

All the patients underwent a comprehensive neurological examination. To evaluate sensory involvement, the inflammatory neuropathy cause and treatment (INCAT) sensory sum score (ISS) ranging from 0 to 20 was obtained [13]. To access the global degree of disability, we obtained the INCAT overall disability sum score (ODSS, ranging from 0 to 10) [14] and inflammatory Rasch-built overall disability scale (RODS, percentiles) [15].

### Electrophysiological assessment

The NCS were conducted using a Dantec^™^ Keypoint^®^ Focus electromyographic (EMG) device (Natus Medical GmbH, Planegg, Germany). Standard techniques were used for percutaneous supramaximal stimulation and surface electrode in standardized conditions with skin temperatures of at least 33 °C at the palm and 30 °C at the external malleolus. Bilateral NCS were performed on the median (motor and sensory), ulnar (motor and sensory), radial (sensory), tibial (motor), fibular (motor and sensory), and sural (sensory) nerves in all patients. The NCSs were performed according to the methodology described by Stöhr et al. [16].

### CCM

All CCM were executed by AA, NS and DS using a Heidelberg Retinal Tomograph III with a Rostock Cornea Module (Heidelberg Engineering GmbH, Heidelberg, Germany). CCM was performed bilaterally as previously described. Both eyes were tested and 5 CCM images per eye were analysed and evaluated including the image quality [17]. The mean value of all quality indices (QI) per eye was calculated. From a QI < 1.8, a manual evaluation with the software CCM-Metrics was performed in addition to a fully automated evaluation with the software ACCM [18] or the freeware Image J with the addon Neuron J [19]. The following nerve parameters were analysed: CNFL [mm/mm^2^], CNFD [number/mm^2^] and CNBD [number/mm^2^].

### Ethical approval

All procedures performed in this study were in accordance with the ethical standard of the institutional and national research committee and with the 1964 Helsinki Declaration and its later amendments. The INHIBIT register was approved by the local ethics committee (vote no. 18–6534-BR, Ruhr-University Bochum, Germany) and was registered in the German register of clinical studies (Deutsches Register Klinischer Studien (DRKS), register name: Immunmediated Neuropathies Biobank INHIBIT; register number: DRKS00024494; registered on 11 February 2021). Healthy controls were recruited from BioNerve study (vote no. 4905-14) and INHIBIT register of the Ruhr-University Bochum, Germany.

### Statistics

The statistical analysis was conducted using IBM^®^ SPSS Statistics (version 27.0.0.0). All data are presented as mean with standard deviation (SD). Nominal and dichotomous variables are presented as counts and percentages. Ordinal variables were presented as median with interquartile ranges (IQR). Demographics and clinical characteristics were compared using Student’s *t*-test for numerical normally distributed variables, Mann–Whitney-U-test for numerical non-normally distributed values, or chi-squared test (χ^2^-test) for nominal variables. Multiple comparisons of more than two groups were performed using Kruskal–Wallis test with post hoc Bonferroni correction. Correlations were performed using Spearman’s rank correlation for non-normally and Pearsons correlation for normally distributed variables. To assess different disease stages, patients were divided into quartiles based on their disease duration (Q1 = very early disease stage, Q2 = early disease stage, Q3 = late disease stage, Q4 = very late disease stage). For all analyses, the statistically significant threshold was set at *p*-value < 0.05.

## Results

### Demographic characteristics and comparability to HC

In total, 100 CIDP patients were analysed. The mean age was 57 ± 11 years and the disease duration was 49 ± 52 months since first manifestation of symptoms. 76% of patients were men. 53/100 patients (53%) suffered from typical CIDP. The remaining patients (47%) suffered from atypical CIDP such as MADSAM (n = 15), DADS (n = 28), and other (n = 4). To normalize the influence of age and gender on the CCM analysis, we used a stratified HC consisting of 31 individuals. The mean age of HC was 52 ± 15 and 65% men were included. There were no significant differences between HC and CIDP patients in age and sex. Sociodemographic details and clinical characteristics are displayed in Table 1.

**Table 1:** sociodemographic data and clinical scores of patients. Stars are visualizing significant differences to healthy controll (HC)

	All CIDP	Typical CIDP	Atypical CIDP	MADSAM	DADS	HC
n (total%)	100 (100)	53 (53)	47 (47)	15 (15)	28 (27)	31
Female (group%)	24 (24)	15 (28)	9 (19)	2 (13)	4 (14)	11 (35)
Male (group%)	76 (76)	38 (72)	38 (81)	13 (87)	24 (86)	20 (65)
Age ± SD (n)	57 ± 11	58 ± 11	56 ± 12	57 ± 11	57 ± 10	52 ± 15
CNFL [mm/mm^2^], mean ± SD (n)	14.6 ± 3.6***	14.6 ± 3.2**	14.5 ± 4.1**	15.1 ± 3.6	13.6 ± 4.1**	18.6 ± 6.9
CNFD [/mm^2^], mean ± SD (n)	24.4 ± 7***	25.4 ± 6.6*	23.2 ± 7.1***	24.2 ± 6.8	22.3 ± 7.7**	29.5 ± 4.9
CNBD [/mm^2^], mean ± SD (n)	30.7 ± 17.5***	30.1 ± 17.4**	31.3 ± 17.7*	34.1 ± 17.6	27.6 ± 17.4**	42.7 ± 18.8
Time since diagnosis, months ± SD	49 ± 52	59 ± 58	38 ± 41	55 ± 60	31 ± 28	
Time since manifestation, months ± SD	82 ± 63	86 ± 65	78 ± 61	99 ± 85	72 ± 44	
Overall ODSS, median (IQR)	3 (2)	3 (2)	2 (1)	3 (2)	2 (2)	
ODSS Arm, median (IQR)	1 (2)	2 (2)	1 (2)	2 (1)	1 (1)	
ODSS Leg, median (IQR)	2 (1)	3 (2)	2 (1)	2 (1)	1 (1)	
R-ODS, mean ± SD	68 ± 19	64 ± 18	73 ± 19	62 ± 21	81 ± 16	
ISS Sum Score, mean ± SD	6.6 ± 4.3	7.3 ± 4.4	5.9 ± 4	6.1 ± 5.2	5.9 ± 3.1	

### Corneal nerve parameters in CIDP: Significant reduction but limited diagnostic utility

**Fig. 1:**
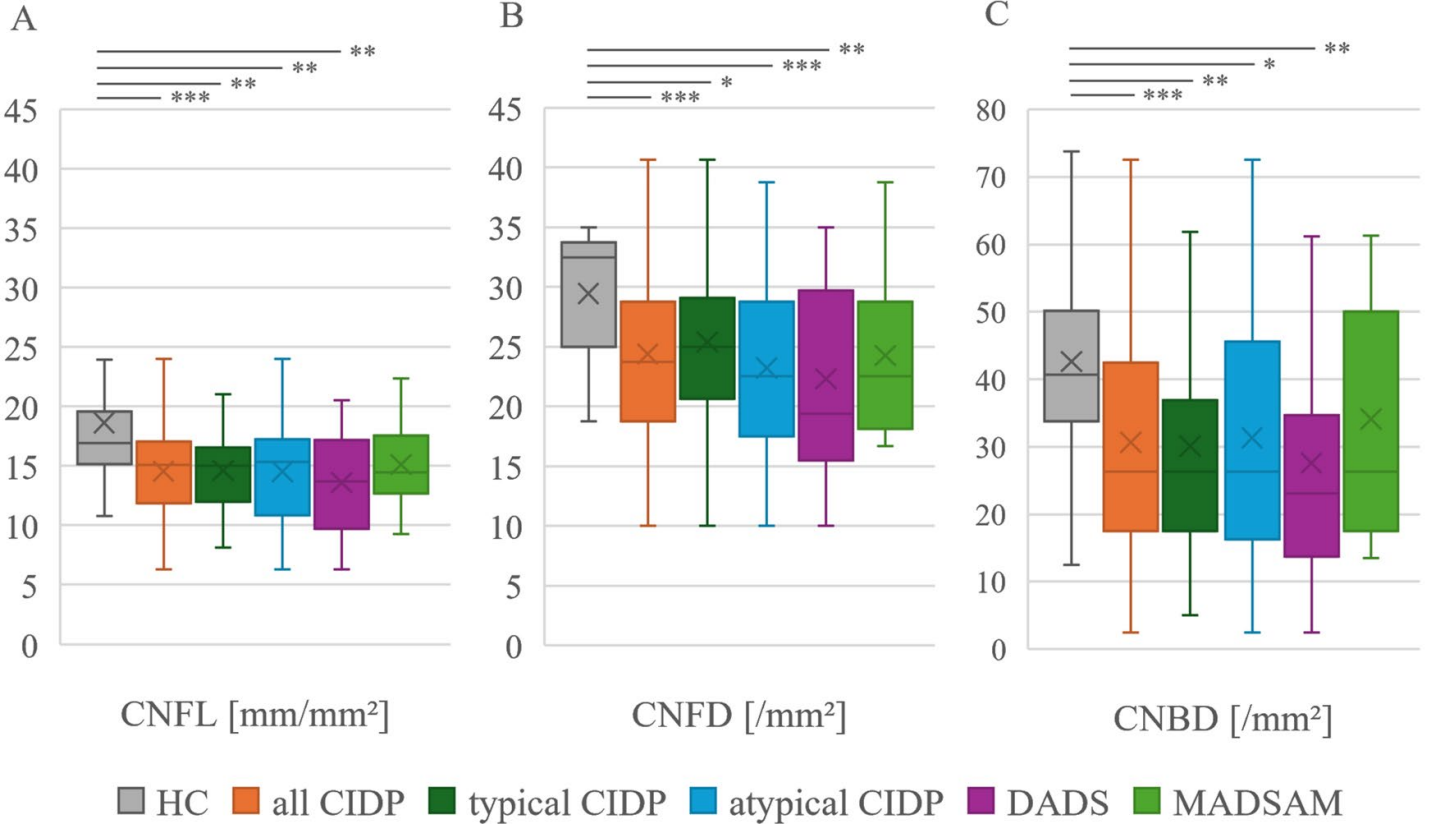
Comparison of CCM parameters in all patients (n = 100), typical (n = 53) and atypical CIDP (n = 47), DADS (n = 28) and MADSAM (n = 15) with HC (n = 31). Values are displayed as mean ± SD. Comparison was performed using Kruskal Wallis test with Bonferonni correction. A: Significant reduction of CNFL in all patients (14.6 ± 3.6; p < 0.001), typical (14.6 ± 3.2; p = 0.002) and atypical CIDP (14.5 ± 4.1; p = 0.006), and DADS (13.6 ± 4.1; p = 0.004). HC = 18.6 ± 6.9. B: Significant reduction of CNFD in all patients (24.4 ± 7; p < 0.001), typical (25.4 ± 6.6; p = 0.028) and atypical CIDP (23.2 ± 7.1; p = 0.001), and DADS (22.3 ± 7.7; p = 0.002). HC = 29.5 ± 4.9. C: Significant reduction of CNBD in all patients (30.7 ± 17.5; p < 0.001), typical CIDP (30.1 ± 17.4; p = 0.003), atypical CIDP (31.3 ± 17.7; p = 0.011), and DADS (27.6 ± 17.4; p = 0.005). HC = 42.7 ± 18.8. No significant differences between typical and atypical CIDP

Comparison of CCM parameters CNFL, CNFD, and CNBD revealed significant reduction in CIDP patients and subgroups compared to HC. All CIDP patients showed a mean CNFL value of 14.6 ± 3.6 while HC showed a value of 18.6 ± 6.9 (p < 0.001). Similar results were observed when comparing CNFL of patients with atypical CIDP (14.6 ± 3.2; p = 0.002), typical CIDP (14.5 ± 4.1; p = 0.006) and DADS (13.6 ± 4.1; p = 0.004) compared to HC. Only MADSAM did not show this pattern (15.1 ± 3.6; p > 0.05). The parameters CNFD and CNBD were significantly reduced as well. Details are displayed in Fig. 1. To assess the diagnostic accuracy of CCM parameters, we evaluated the sensitivity and specificity of each individual CCM parameter and all CCM parameters combined, using previously published age- and sex-adjusted CCM normative values [14]. In the combined evaluation of sensitivity and specificity, a positive result was assumed if at least one of the CCM parameters was below the adjusted normal values. In combination, CCM parameters reached a sensitivity of 41% and a specificity of 77%. Details of the evaluation are presented in Table 2.

**Table 2:** Sensitivity and specificity of the individual CCM parameters as well as in combination**.** Used corneal nerve normative values were published 2015 by Tavakoli et. al

CNFL	CIDP	HC	Sensitivity (%)	Specificity (%)
Positive CCM result	36	6	36%	81%
Negative CCM result	64	25		
**CNFD**	**CIDP**	**HC**	**Sensitivity (%)**	**Specificity (%)**
Positive CCM result	4	0	4%	100%
Negative CCM result	96	31		
**CNBD**	**CIDP**	**HC**	**Sensitivity (%)**	**Specificity (%)**
Positive CCM result	22	5	22%	84%
Negative CCM result	78	26		
**Combined**	**CIDP**	**HC**	**Sensitivity (%)**	**Specificity (%)**
Positive CCM result	41	7	41%	77%
Negative CCM result	59	24		

### Association between CCM parameter severity and disease progression in CIDP

**Fig. 2:**
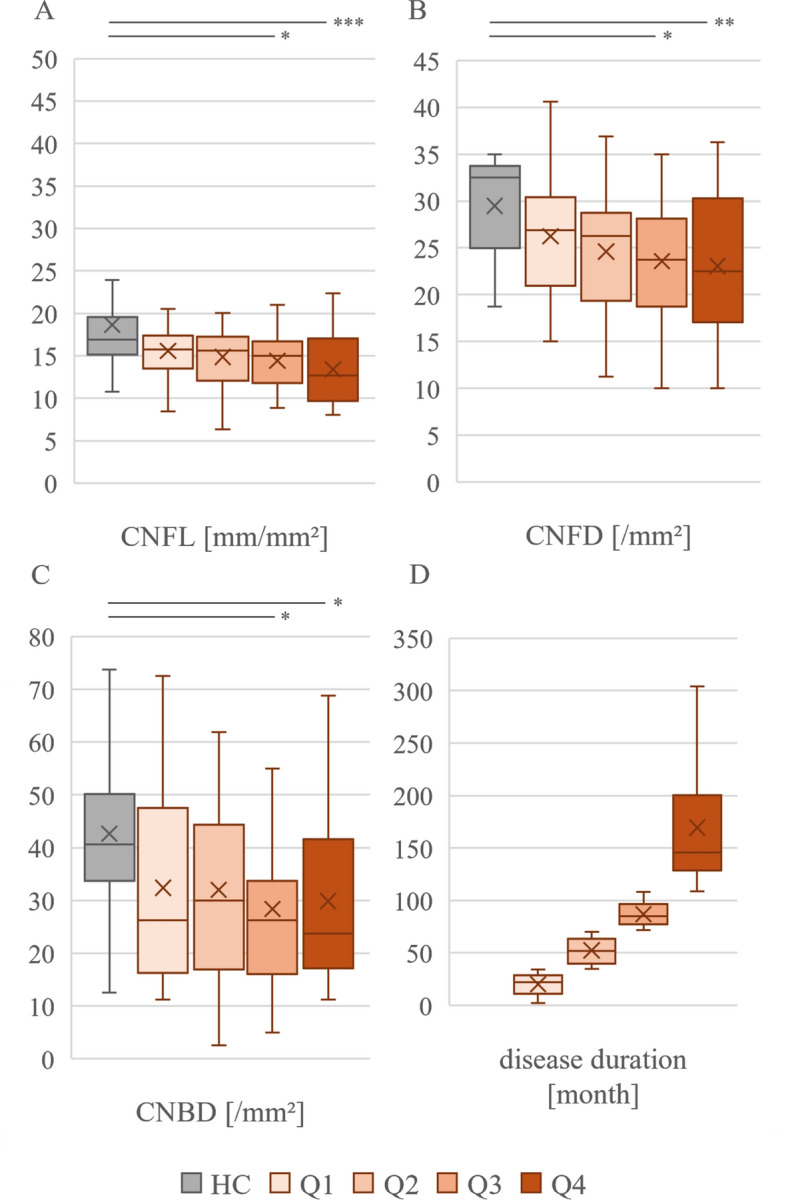
Comparison of different disease stages of all CIDP (Q1–Q4; n = 25/group) patients with HC (n = 31). Values are displayed as mean ± SD. Comparison was performed using Kruskal Wallis test with Bonferonni correction. A: Significant reduction of CNFL in Q3 (14.4 ± 3.0; p = 0.025) and Q4 (13.4 ± 4.1; p = 0.001) compared to HC (18.6 ± 6.9). B: Significant reduction of CNFD in Q3 (23.6 ± 5.9; p < 0.022) and Q4 (23.0 ± 7.6; p = 0.007) compared to HC (29.5 ± 4.9). C: Significant reduction of CNBD in Q3 (28.4 ± 18.0; p < 0.001) and Q4 (29.9 ± 17.1; p = 0.005) compared to HC = 42.7 ± 18.8. D: Disease durations since first manifestation in month mean ± SD. Q1 = 20 ± 10; Q2 = 53 ± 15; Q3 = 87 ± 11; Q4 = 169 ± 58

We pairwise compared different disease stages with HC by dividing patients into quartiles based on their disease duration. Disease duration in Q1 was 20 ± 10 months (n = 25), in Q2 53 ± 15 months (n = 25), in Q3 87 ± 11 months (n = 25), and in Q4 169 ± 58 months (n = 25) in all CIDP patients. Comparing CCM measurements of these disease stages with HC revealed significant reduction of CNFL (Q3: 14.4 ± 3.0, p = 0.025; Q4: 13.4 ± 4.1, p < 0.001), CNFD (Q3: 23.6 ± 5.9, p = 0.022; Q4: 23.0 ± 7.6, p = 0.007), and CNBD (Q3: 28.4 ± 18.0, p = 0.014; Q4: 29.9 ± 17.1, p = 0.046) at late (Q3) and very late (Q4) disease stage. Details are displayed in Fig. 2. Similar results were obtained in the comparison of typical CIDP and atypical CIDP with HC (supplementary Table 1).

**Fig. 3:**
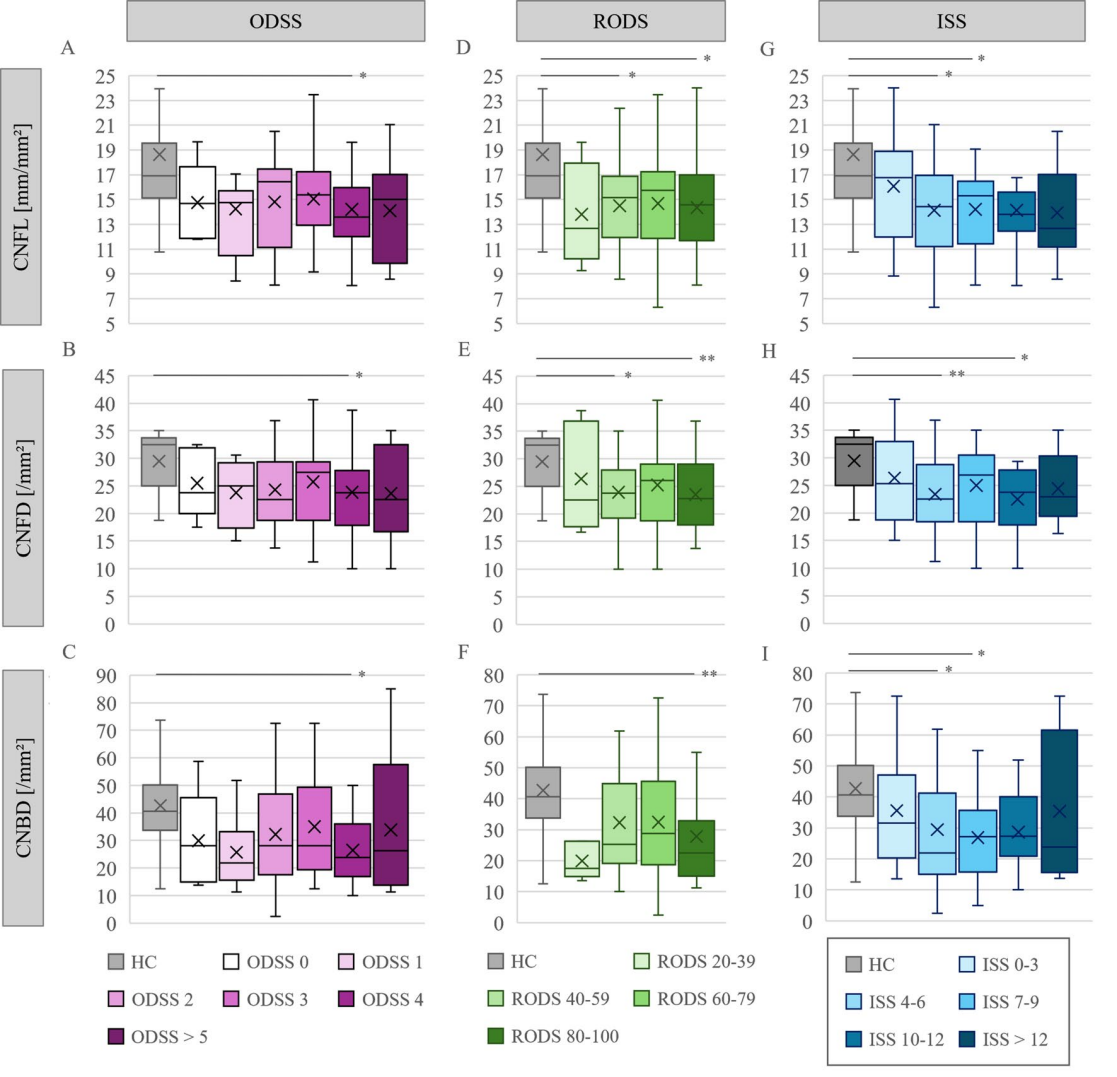
Comparison of CCM parameters with clinical scores of all CIDP patients with HC (n = 31). Comparison was performed using Kruskal Wallis test with Bonferonni correction. Significant reduction of CNFL (A: 14.2 ± 3.3, p = 0.004), CNFD (B: 23.8 ± 7.2, p = 0.014) and CNBD (C: 28.6 ± 16.3, p = 0.015) in patients with ODSS > = 4. Patients with RODS 76–100 showed significant reduction of CNFL (D: 14.2 ± 3.6, p = 0.003), CNFD (E: 23.3 ± 6.1, p = 0.001), and CNBD (F: 27.7 ± 15.9, p = 0.001). Patients with RODS of 51–75 showed significant reduction of CNFL (**D:** 14.5 ± 3.4, p = 0.009) and CNFD (**E:** 25.0 ± 7.1, p = 0.049). Patients with ISS sum score > = 11 showed significant reduction of CNFL (G: 14.1 ± 3.6, p = 0.002) and CNFD (H: 23.6 ± 6.1, p = 0.007). Patients with ISS sum score of 6 to 10 showed significant reduction of CNFL (G: 14.1 ± 3.1, p = 0.002), CNFD (H: 24.1 ± 6.8, p = 0.012), and CNBD (I: 27.9 ± 14.1, p = 0.004). Patients with ISS sum score of 0 to 5 showed significant reduction of CNFD (H: 24.8 ± 7.1,p = 0.019) and CNBD (I: 32.5 ± 19.0, p = 0.043). ODSS 0: n = 5; ODSS 1: n = 12; ODSS 2: n = 29, ODSS 3: n = 19; ODSS > = 4: n = 35. RODS 26–50: n = 21, RODS 51–75: n = 38, RODS 76–100: n = 37. ISS 0

Furthermore, we performed a more detailed analysis of the relationship between clinical scores (ODSS, RODS, and ISS sum score) and CCM parameters. For this purpose, we divided the patients into subgroups based on their scores and compared them to HC. To ensure comparable group sizes, patients with an ODSS > = 4 or an ISS sum score > = 11 were pooled. Results are displayed in Fig. 3. Patients with an ODSS > = 4 exhibited a significant reduction in all CCM parameters (Fig. 3 A-C. CNFL: p = 0.004. CNFD: p = 0.014, CNBD: p = 0.015). This association was not observed for lower ODSS scores. A similar finding was observed for the ISS sum score. Patients with an ISS sum score > = 11 showed a significant loss of CNFL (Fig. 3G, p = 0.014) and CNFD (Fig. 3H, p = 0.041) while patients with a ISS of 6–10 showed significant reduction in all CCM parameters (CNFL: p = 0.002. CNFD: p = 0.012. CNBD: p = 0.004) compared to healthy controls. Patients with low ISS of 0–5 showed a significant reduction of CNFD (p = 0.019) and CNBD (p = 0.043) as well.

Evaluation of the RODS revealed a different pattern, with lower CNFL (Fig. 3D, p = 0.002), CNFD (Fig. 3E, p = 0.007), and CNBD (Fig. 3E,, p = 0.001) observed particularly in patients with high scores of 76–100 and, therefore, better functional status. However, patients with a RODS value of 51–75 had significant lower CNFL (Fig. 3D, p = 0.009) and CNFD (Fig. 3E, p = 0.049), too.

### CCM parameters and SNAPs as complementary markers for nerve fiber integrity in CIDP patients

**Fig. 4:**
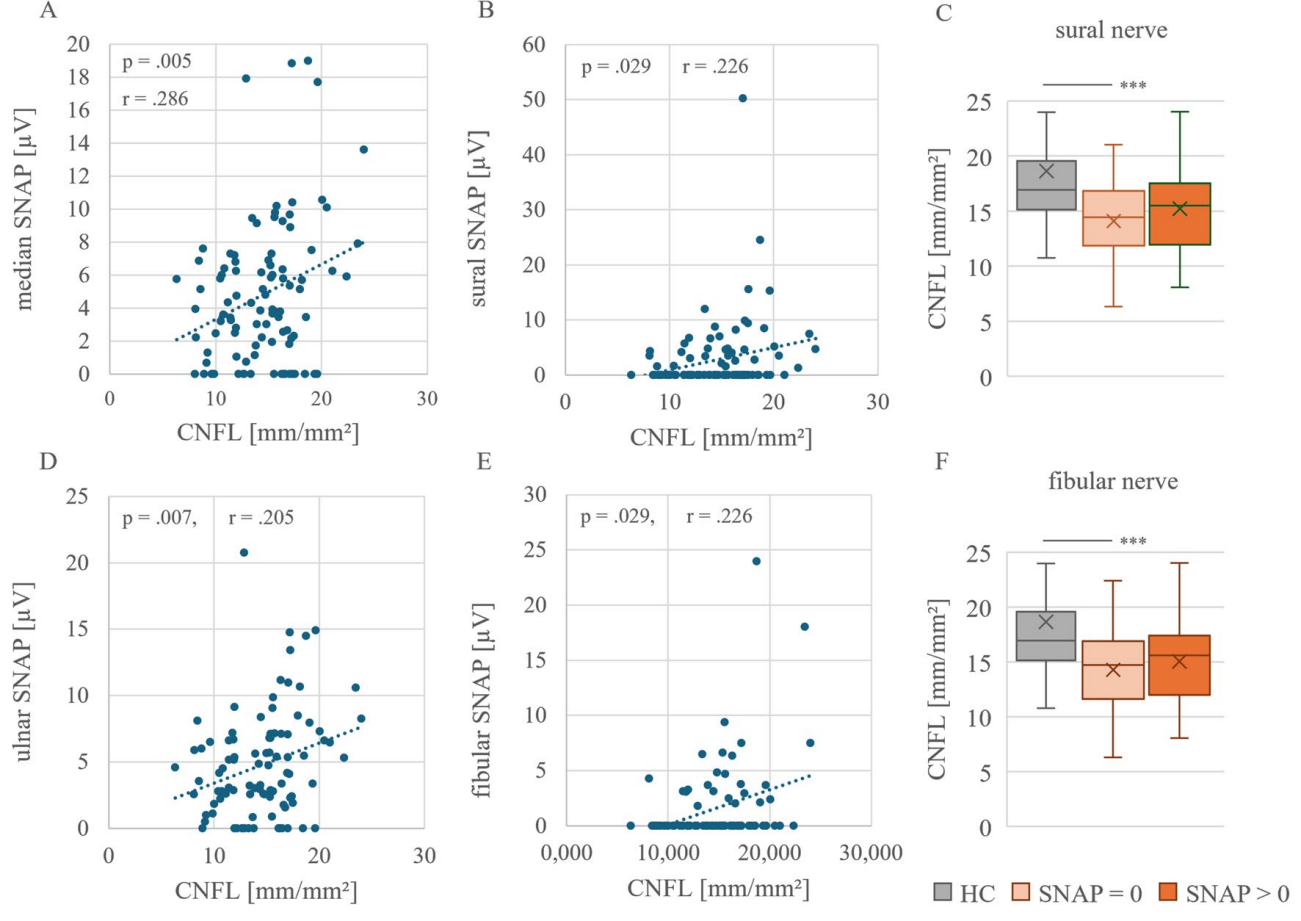
Pearson correlations of CNFL with SNAP of median (A), sural (B), ulnar (D), and fibular (E) nerve as well as comparison of CNFL in patients with and without extinguished sural (C) and fibular (F) nerve compared to HC (n = 31). CNFL showed significant weak positive correlations with SNAP of median, sural, ulnar, and fibular nerve. Besides, significant reduction of CNFL in patients with extinguished sural (C: 14.1 ± 3.3, p < 0.001) and fibular (F: 14.3 ± 3.3, p < 0.001) nerve compared to HC (18.6 ± 6.9). Sural SNAP = 0 µV (n = 31). Sural SNAP > 0 µV (n = 36, CNFL: 15.2 ± 3.9). Fibular SNAP = 0 µV (n = 61). Fibular SNAP > 0 µV (n = 24, CNFL: 15.1 ± 3.7)

In addition to clinical parameters, we also investigated the correlation between sensory nerve action potentials (SNAPs) of the ulnar, median, radial, sural, and fibular nerves with CCM parameters. The mean of both amplitude sides was always used for the analysis. The median nerve was measured in 96 of the 100 patients. 94 measurements of the ulnar nerve were available. 94 measurements of the sural nerve and 85 measurements of the fibular nerve were recorded. The correlation graphs can be seen in Fig. 4.

The Pearson correlation of the measured SNAPs with the CCM parameters showed a significant slight positive correlation of CNFL with the median nerve (Fig. 4A: r = 0.286, p = 0.05), sural nerve (Fig. 4B: r = 0.226, p = 0.029), the ulnar nerve (Fig. 4C: r = 0.277, p = 0.007), and the fibular nerve (Fig. 4D: r = 0.311, p = 0.004). Additionally, significant correlations were found with CNFD and the ulnar and fibular nerves, and CNBD with the median, ulnar, and fibular nerves (supplementary Table 2). In addition to Pearson correlation, we investigated whether patients with absent sural or fibular SNAP had lower CNFL values than patients with preserved amplitude. A significant reduction in CNFL was observed in patients with absent sural (Fig. 4C: 14.1 ± 3.1, p < 0.001) and fibular (Fig. 4C: 14.2 ± 3.4, p = < 0.001). SNAP compared to HC. This association was not detectable in patients with preserved SNAP (Sural SNAP > 0 µV: 15.2 ± 3.9, p = 0.058. Fibular SNAP > 0 µV: 15.0 ± 3.7, p = 0.118).

We also investigated the correlation between CMAP amplitude and CCM parameters. However, no significant correlation was found (supplementary Table 2).

## Discussion

Precise therapy monitoring to prevent secondary damage is a key problem in chronic immune-mediated neuropathies. Recent studies have already identified CCM as a method for monitoring changes in the corneal nerve plexus and CICs [9, 12, 20]—its non-invasiveness and ease of application emphasize the usefulness of the method in this context.

In this study, we demonstrate the ability of CCM to detect degenerative axonal damage. Firstly, our data show that CCM can differentiate between healthy controls and CIDP-patients. Furthermore, it is even possible to distinguish between CIDP subgroups (typical CIDP, atypical CIDP, and DADS) and healthy subjects. Interestingly, no significant differences between MADSAM and healthy controls were observed, which might be due to the small sample size.

Overall, these results are in line with previously published studies on this topic [9]. Although many studies support the usefulness of CCM in diagnosing peripheral neuropathy [9, 21], the diagnostic accuracy of this technique in CIDP has not yet been investigated. Therefore, previously published age- and sex-dependent reference values [22] were used to calculate the sensitivity and specificity of the method. However, in this context the results are ambiguous. While CCM was able to distinguish CIDP patients from healthy subjects in our cohort, when applying the reference values, the sensitivity was only 41% and the specificity was 77% when considering different axonal corneal nerve parameters combined. CNFD alone has a specificity of 100% and might therefore be considered an exclusion marker for CIDP.

One potential reason for the low sensitivity and specificity might be due to the reduction of CCM parameters predominantly in the later stages of the disease. Since these disease stages are less characterized by neuroinflammation but rather by neurodegeneration, CCM might primarily correlate with signs of axonal degeneration which weakens its value as a diagnostic tool. Especially early disease stages are predominantly characterized by peripheral neuroinflammation. However, no correlation of CCM parameters and clinical scores was found for early stages of this disease. These results might emphasize the role of CCM for monitoring disease progression and axonal pathology. Patients with a higher ODSS showed a significantly lower level of axonal CCM parameters compared to healthy controls. Similarly, lower CCM parameters were observed with an increased ISS sum score, which reflects a pronounced sensory impairment. However, the RODS, which represents daily functional activities, stands out from these observations. Although these scores show some associations with the CCM parameters, a significant correlation between the values was not observed. This suggests that the relationship between the clinical scores and the axonal damage measured by CCM is not linear.

In addition to clinical evidence that CCM primarily detects later stages of CIDP, there is also electrophysiological evidence that it may primarily depict the axonal integrity of the nerves. Our data shows that most axonal CCM parameters correlate positively with the SNAP amplitude of the median, ulnar, sural, and fibular nerves. The reduction in CNFL, CNFD and CNBD is therefore directly related to the sensory axons of the peripheral nerves. In contrast, no correlations with CMAP amplitude (motor evoked potentials) were found. CCM measures the nerve endings of the trigeminal nerve in the cornea [23], which are assumed to be Aδ- and C-fibers. Therefore they transmit somatosensory afferent information, leading CCM to be considered a tool for the evaluation of small fiber integrity, like previous studies have already shown and which might be the link to our electrophysiological results [24–26]. We have also shown that CCM parameters were significantly reduced in patients with absent sural and fibular SNAP, detecting an axonal damage in sensory nerves in particular. Therefore, it can be concluded that the method allows more than just the evaluation of small fibers integrity.

Like other chronic neuroimmunological diseases, CIDP is likely to transition from an inflammatory to a neurodegenerative phase, with the inflammatory phase having a better response to therapies. In particular, sensitive nerves are also involved here. The results shown and the linking of corneal and electrophysiological findings could help to recognize this progress of the disease at an earlier stage and, if necessary, to adapt the therapy. However, this hypothesis needs to be confirmed through further longitudinal studies and axonal damage in electrophysiological measurements determine directly clinical disability [27].

Our study also has some limitations. While the chosen quartiles for disease stages are a useful tool, they also reduce the sample size for analysis. A longitudinal follow-up of CIDP patients from the time of first diagnosis would be adequate to evaluate the role of CCM primarily for the evaluation of axonal degeneration.

## Supplementary Information

Below is the link to the electronic supplementary material.

Supplementary file1 (PNG 73 KB)

Supplementary file2 (PNG 59 KB)

## Data Availability

Data collected from this study are available by emailing rafael.klimas@rub.de.

## Declarations

### Patient and public involvement

The patients gave spoken and written consent for publication and participation to the study.
