# Supplementary figures and images for: Assessing axonal pathology and disease progression in chronic inflammatory demyelinating polyneuropathy using corneal confocal microscopy
Source: J Neurol. 2024 Dec 12;272(1):51. doi: 10.1007/s00415-024-12812-4 (PMC11638281; doi:10.1007/s00415-024-12812-4)

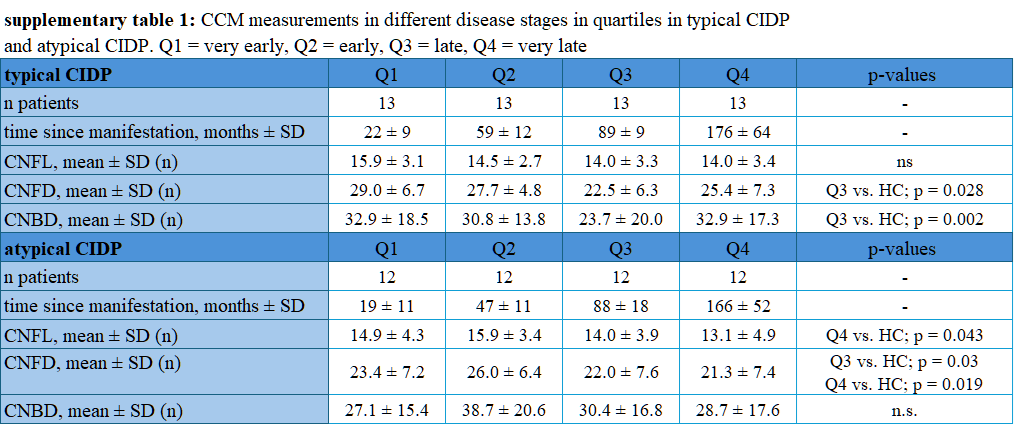

Supplement: Supplementary file 1 — Supplementary file1 (PNG 73 KB) [file 415_2024_12812_MOESM1_ESM.png]

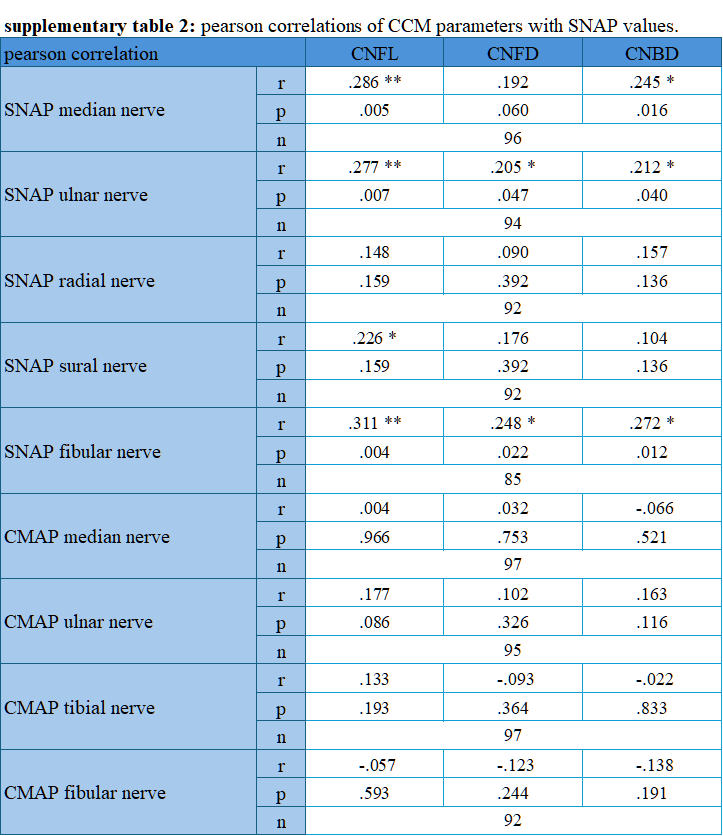

Supplement: Supplementary file 2 — Supplementary file2 (PNG 59 KB) [file 415_2024_12812_MOESM2_ESM.png]
